# Supplementary material for: Attenuation Coefficient Estimation for PET/MRI With Bayesian Deep Learning Pseudo-CT and Maximum-Likelihood Estimation of Activity and Attenuation
Source: IEEE Trans Radiat Plasma Med Sci. Author manuscript; Available in PMC 2024 Jan 12. (PMC10785227; doi:10.1109/trpms.2021.3118325)
Supplement: supp1-3118325 [file NIHMS1820852-supplement-supp1-3118325.pdf]

Supplementary Material for “Attenuation Coefficient Estimation for PET/MRI With Bayesian Deep Learning pseudo-CT and Maximum Likelihood Estimation of Activity and Attenuation” by A. P. Leynes et al.

Sources of uncertainty and variations

There are three different predictive uncertainties that are utilized in our work: total voxel uncertainty--and its components--patch uncertainty, and voxel-wise uncertainty.

Total voxel uncertainty is the combination of patch uncertainty (uncertainty due to changes in input patch) and uncertainty of each voxel for the same input patch (uncertainty due to changes in the model). These can be decoupled and independently estimated.

Patch uncertainty comes from variations of the response of the CNN due to changes in the input data. Whereas voxel uncertainty (for the same input patch) come from variations of the network parameters with respect to the same input. Mathematically, the predictive likelihood for a single voxel can be written completely as follows:

$$p(y_i^* \mid \boldsymbol{X}^*, \boldsymbol{X}, \boldsymbol{Y}) = \frac{1}{N} \sum_{x^* \in \boldsymbol{X}^*} \int p(y^* | x^*, \theta) p(\theta | \boldsymbol{X}, \mathbf{Y}) \mathrm{d}\theta$$

where y_i^* is the predicted value at the i-th voxel, $\boldsymbol{X}^*$ is the set of neighboring and overlapping input patches, N is the number of patches to predict the value of a single voxel, θ are the network parameters, $\boldsymbol{X}, \boldsymbol{Y}$ are the training input/output pairs, and $p(\theta|\boldsymbol{X}, \mathbf{Y})$ is the posterior distribution of the network parameters given the training pairs that is learned during model training.

The final predicted value is obtained by taking the expectation of the model predictions over the predictive likelihood:

$$\widehat{y_i^*} = E[y_i^*] = \int y_i^* \left(\frac{1}{N} \sum_{x^* \in \boldsymbol{X}^*} \int p(y^* | x^*, \theta) p(\theta | \boldsymbol{X}, \mathbf{Y}) \mathrm{d}\theta \right) dy_i^*$$

$$\approx \frac{1}{NM} \sum_{x^* \in \boldsymbol{X}^*} \sum_{m=1}^{M} y_{i,m}^* \quad (By\ Monte - Carlo\ approximation\ of\ the\ integrals)$$

$$= \frac{1}{NM} \sum_{x^* \in \boldsymbol{X}^*} \sum_{m=1}^{M} f_{CNN}(x^*, \theta_m)$$

And the variance is:

$$\sigma_{y_i^*}^2 = E[(y_i^* - \widehat{y_i^*})^2] = \int (y_i^* - \widehat{y_i^*})^2 \left(\frac{1}{N} \sum_{x^* \in \boldsymbol{X}^*} \int p(y^* | x^*, \theta) p(\theta | \boldsymbol{X}, \mathbf{Y}) \mathrm{d}\theta \right) dy_i^*$$

$$\approx \frac{1}{NM} \sum_{x^* \in \boldsymbol{X}^*} \sum_{m=1}^{M} \left(y_{i,m}^* - \widehat{y_i^*} \right)^2 \quad (By\ Monte - Carlo\ approximation\ of\ the\ integrals)$$

$$= \frac{1}{NM} \sum_{x^* \in \boldsymbol{X}^*} \sum_{m=1}^{M} (f_{CNN}(x^*, \theta_m) - \widehat{y_i^*})^2$$

where M is the number of Monte-Carlo samples used in inference.

Voxel uncertainty corresponds to the following term in the predictive likelihood:

$$\int p(y^*|x^*,\theta)p(\theta|\boldsymbol{X},\mathbf{Y})\mathrm{d}\theta$$

and corresponds to the following summation in the prediction and variance:

$$\sum_{m=1}^{M} f_{CNN}(x^*,\theta_m)$$

$$\sum_{m=1}^{M} (f_{CNN}(x^*,\theta_m) - \widehat{y_i^*})^2$$

The patch uncertainty comes from averaging the predictions of different input patches for each single voxel and corresponds to the summation in the prediction and variance:

$$\sum_{x^*\in\boldsymbol{X}^*} f_{CNN}(x^*,\theta_m)$$

$$\sum_{x^*\in\boldsymbol{X}^*} (f_{CNN}(x^*,\theta_m) - \widehat{y_i^*})^2$$

Suppose that there is no model uncertainty, and the network parameters are fixed to be $\hat{\theta}$ (only one set of network parameters used in all inferences). The predictive likelihood will then be

$$p(y_i^*|\,\boldsymbol{X}^*,\boldsymbol{X},\boldsymbol{Y}) = \frac{1}{N}\sum_{x^*\in\boldsymbol{X}^*} p\left(y^*\middle|x^*,\hat{\theta}\right)$$

And the final predicted value will be:

$$\widehat{y_i^*} = \frac{1}{N}\sum_{x^*\in\boldsymbol{X}^*} f_{CNN}\left(x^*,\hat{\theta}\right)$$

And the variance will be

$$\sigma_{y_i^*}^2 = \frac{1}{N}\sum_{x^*\in\boldsymbol{X}^*}\left(f_{CNN}\left(x^*,\hat{\theta}\right) - \widehat{y_i^*}\right)^2$$

Suppose that we do not process overlapping patches and only extract voxel uncertainty, the predictive likelihood will be:

$$p(y_i^*|\,x^*,\boldsymbol{X},\boldsymbol{Y}) = \int p(y^*|x^*,\theta)p(\theta|\boldsymbol{X},\mathbf{Y})\mathrm{d}\theta$$

And the final predicted value will be:

$$\widehat{y_i^*} = \frac{1}{M}\sum_{m=1}^{M} f_{CNN}(x^*,\theta_m)$$

And the variance will be:

$$\sigma^2_{y_i^*} = \frac{1}{M}\sum_{m=1}^{M}(f_{CNN}(x^*, \theta_m) - \widehat{y_i^*})^2$$

Thus, patch uncertainty and (patch-specific) voxel uncertainty can be independently obtained but are tightly coupled together when calculating total voxel uncertainty. In the final prediction for this work, we utilize total voxel uncertainty that incorporates both patch uncertainty and (patch-specific) voxel uncertainty.

Additional information regarding the weight map empirical transformation

The empirical transformation was a heuristic developed for this work to be compatible with the MLAA algorithm of Ahn et al [1]. In the work of Ahn et al, the weight prior, β_{MR}, was defined only by two discrete values: one for the implant region and one for outside the implant region. Our work maintained the use of upper and lower bounds and we used a smooth curve that allows for smoothness as the input values goes towards the saturation values. The predicted variance values have a range of $[0, \infty)$. We set an upper-bound threshold for variance values based on the observed ranges and visual inspection of which anatomic structures the high-variance regions corresponded to. The complete transformation function then has the following requirement: $f: [0, \sigma^2_{max}] \rightarrow [\beta_{MR_{min}}, \beta_{MR_{max}}]$ and we desired a smooth transition towards the saturation values. Thus, we chose a sigmoid function for initial transformation the transformation of the variance values: $f: [0, \sigma^2_{max}] \rightarrow [0, 1]$, and the linear transformation re-maps it to the range needed for β_{MR}: $g: [0, 1] \rightarrow [\beta_{MR_{min}}, \beta_{MR_{max}}]$.

References:

[1] S. Ahn *et al.*, "Joint estimation of activity and attenuation for PET using pragmatic MR-based prior: application to clinical TOF PET/MR whole-body data for FDG and non-FDG tracers," *Phys. Med. Biol.*, vol. 63, no. 4, p. 045006, 2018, doi: 10.1088/1361-6560/aaa8a6.

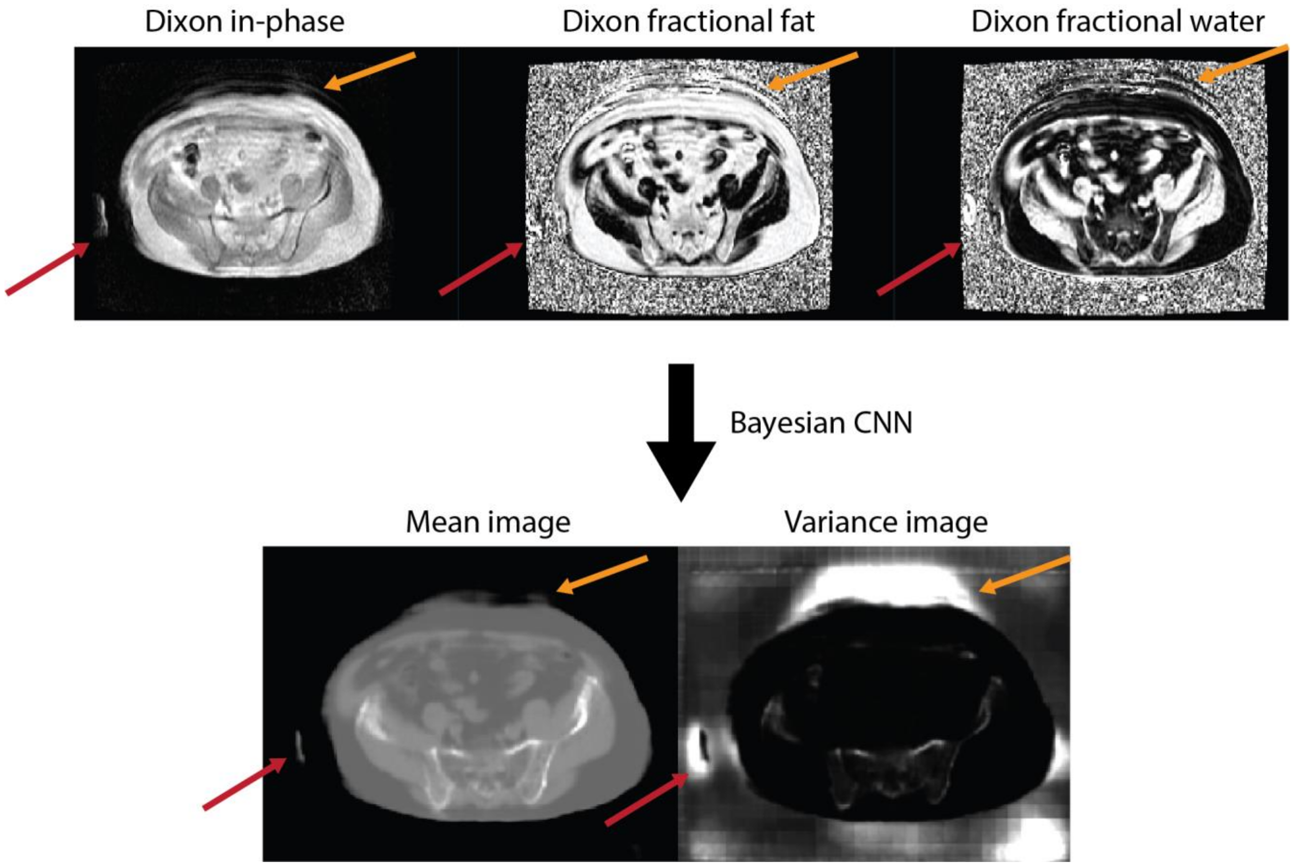

Supp. Fig. 1. Result of uncertainty estimation on without body masks on an MRI with motion artifacts due to breathing and arm truncation due to edge of field-of-view inhomogeneity. The orange arrow points to a region where motion artifacts were present and the dark red arrow points to a region with arm truncation. Both artifact regions were highlighted in the variance image without the network being explicitly trained to highlight these regions.

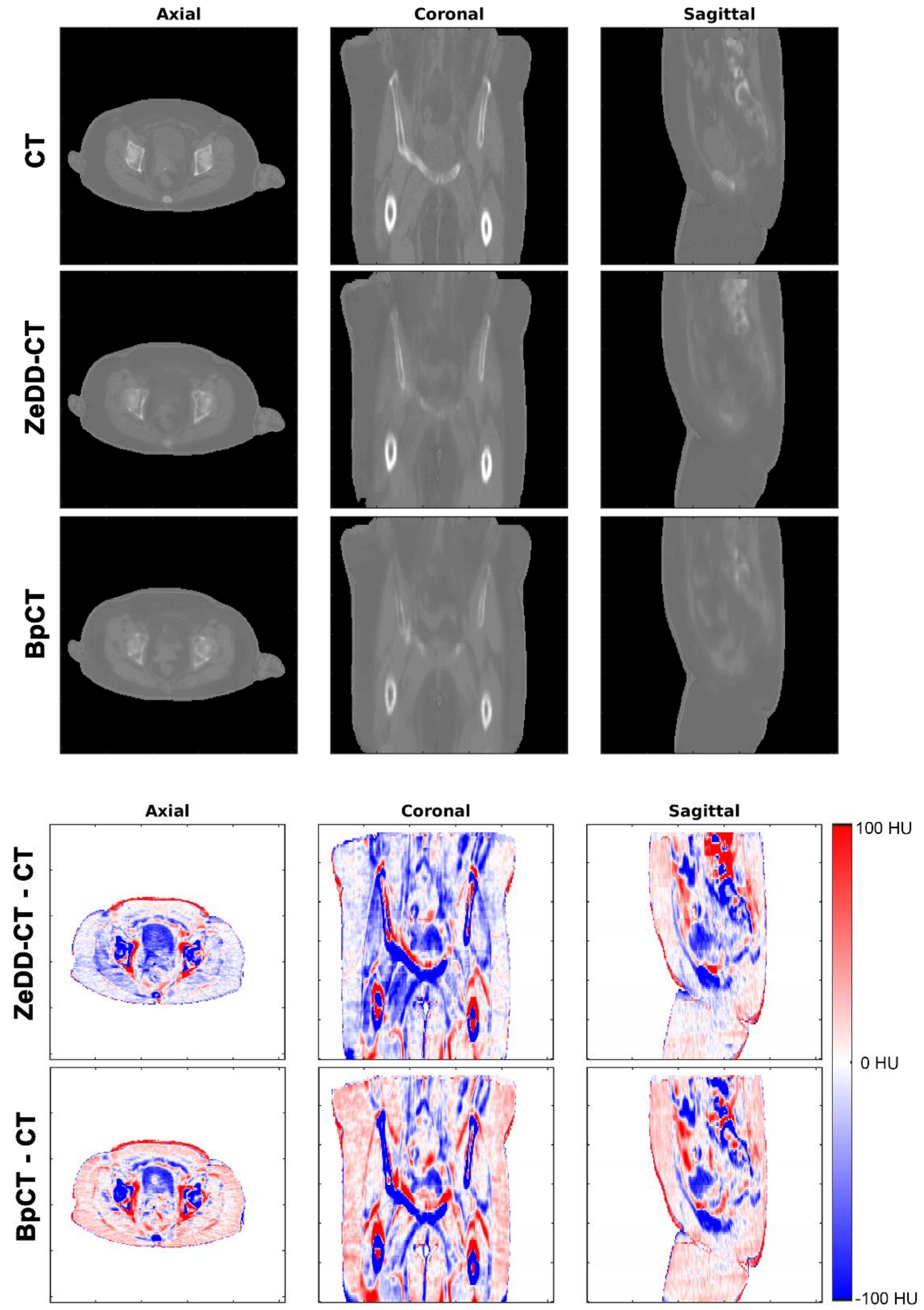

Supp. Fig. 2. CT and difference images of pseudo-CT images for one representative case without implants. Both ZeDD-CT and BpCT have the least RMSE for this patient (RMSE = 98.5 HU, and RMSE = 98.9 HU, respectively).

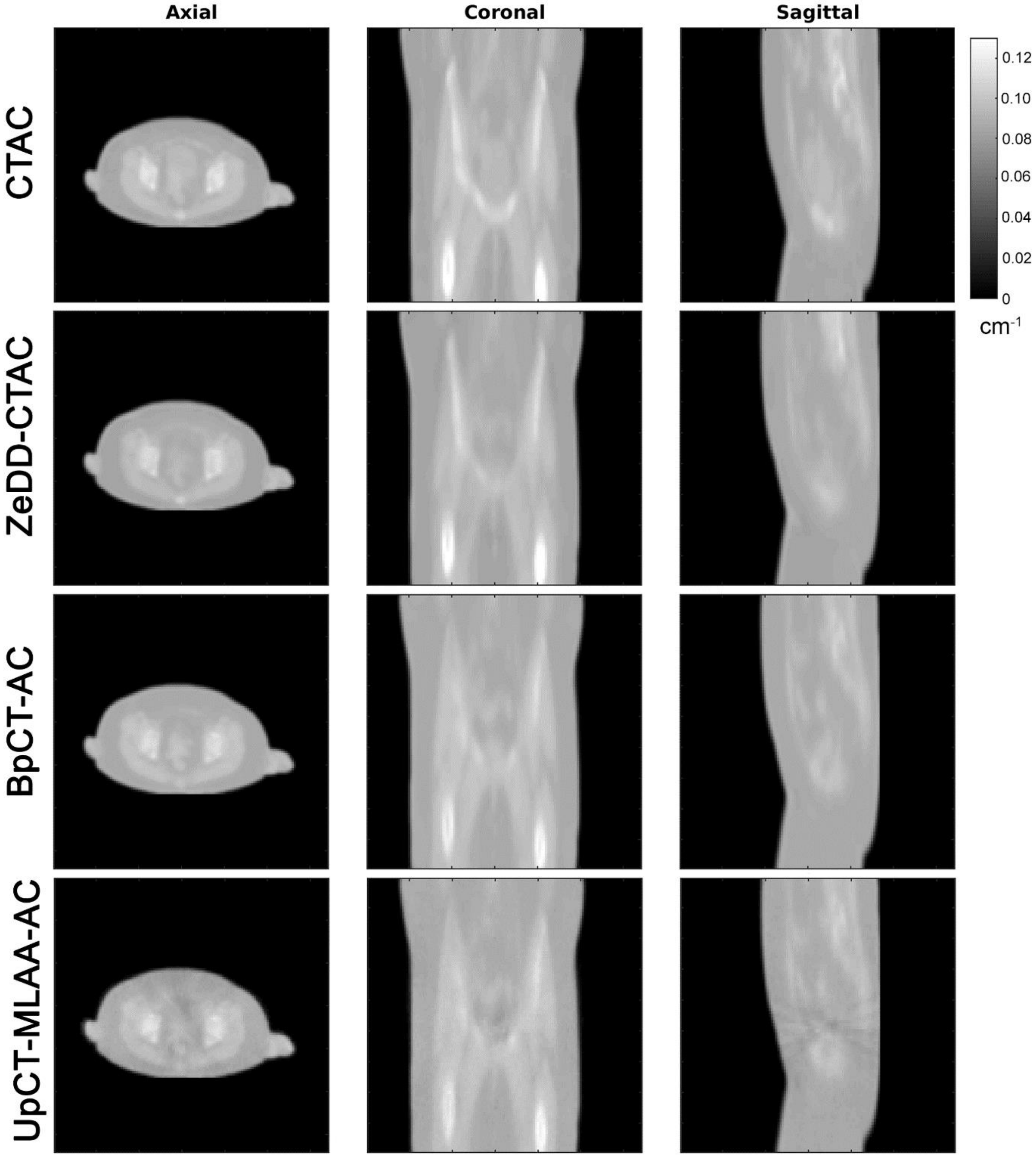

Supp. Fig. 3. CTAC and the different AC maps produced from the different methods for one representative case without implants.

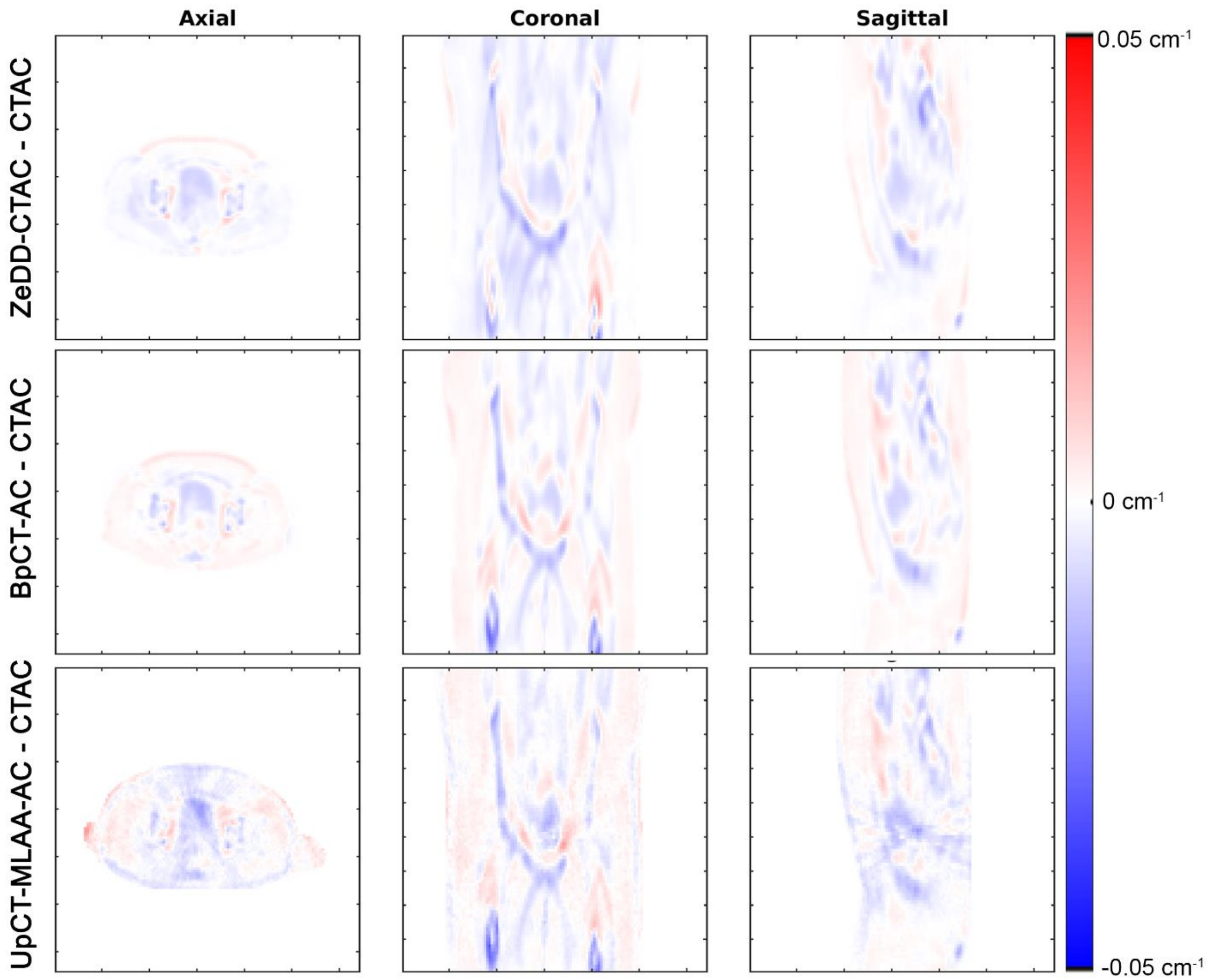

Supp. Fig. 4. Difference images of the AC methods compared to ground-truth CTAC for one representative case without implants.

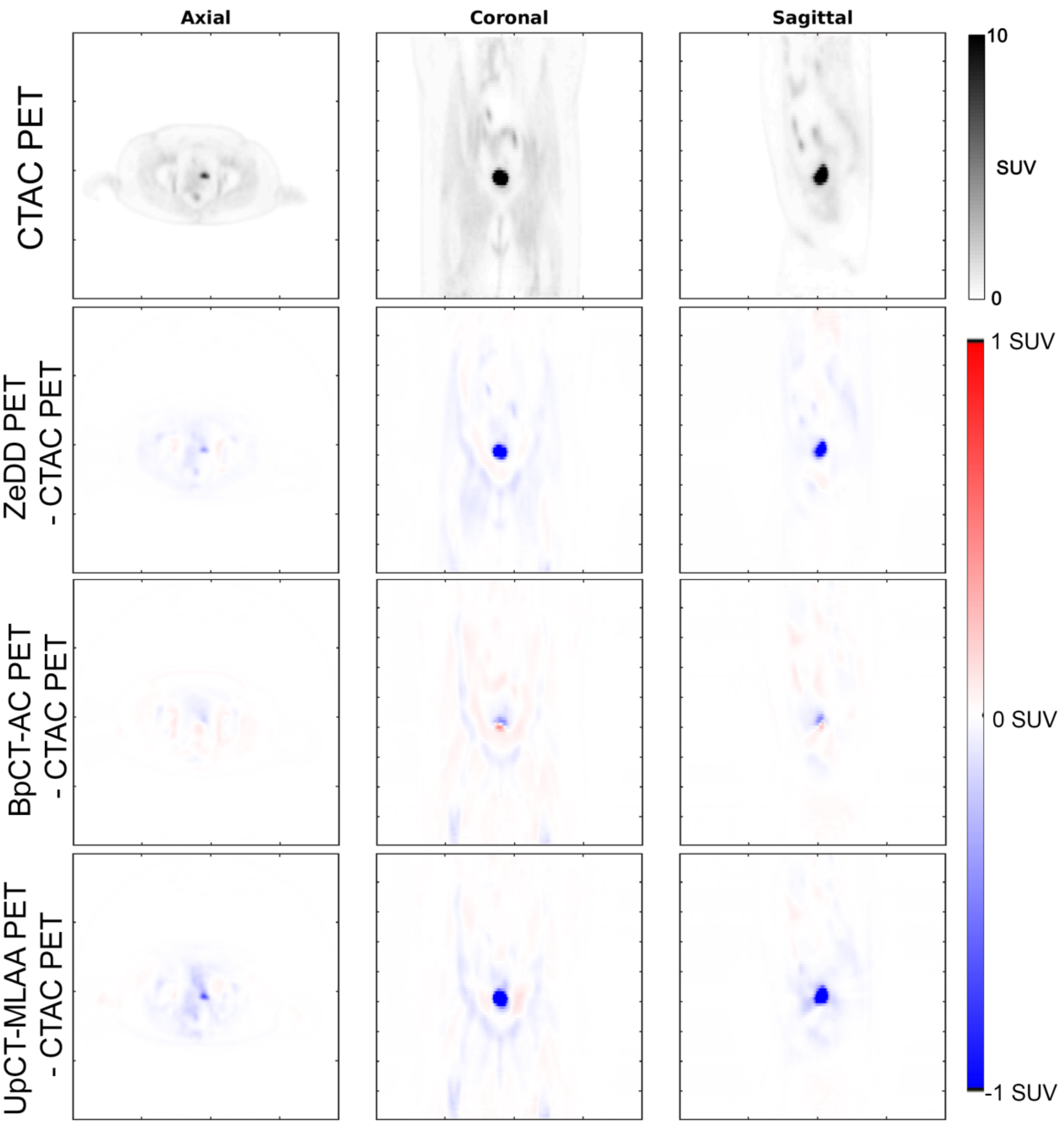

Supp. Fig. 5. PET images of CTAC PET and difference images of the AC methods compared to CTAC PET for one representative case without implants.

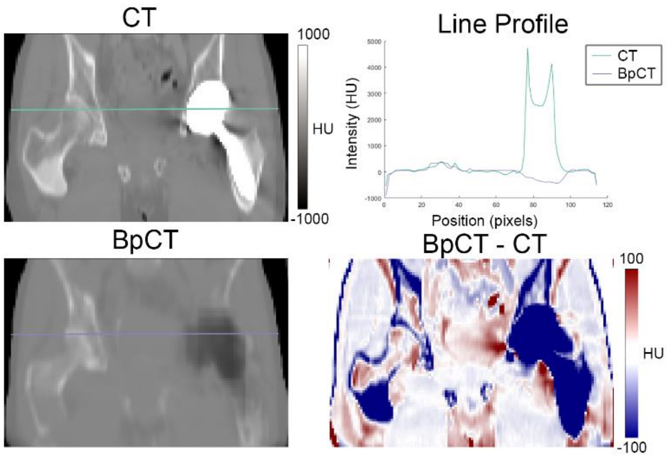

Supp. Fig. 6. CT, pseudo-CT, line profiles, and difference images for a patient with a metal implant imaged with PSMA.

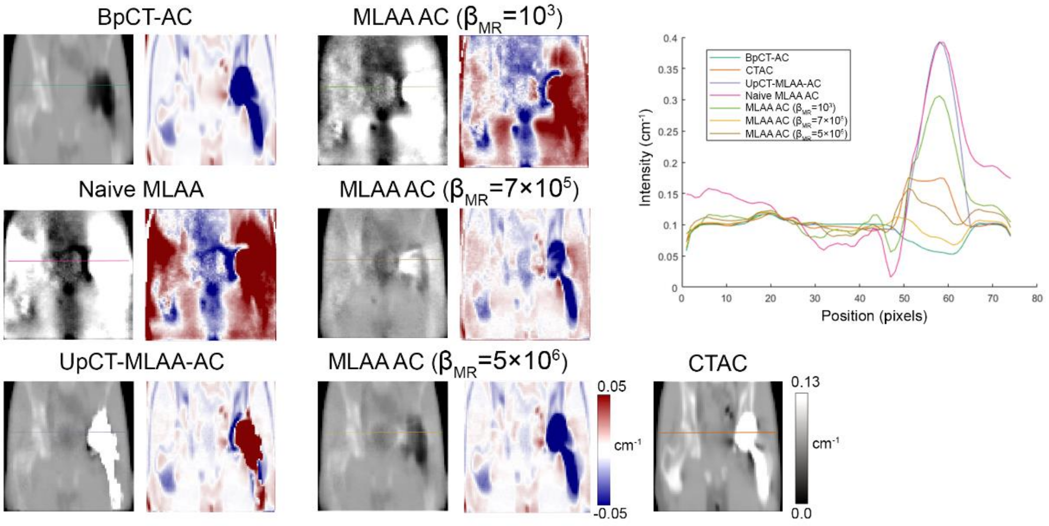

Supp. Fig. 7. AC maps, line profile, and difference images for a patient with a metal implant imaged with PSMA.

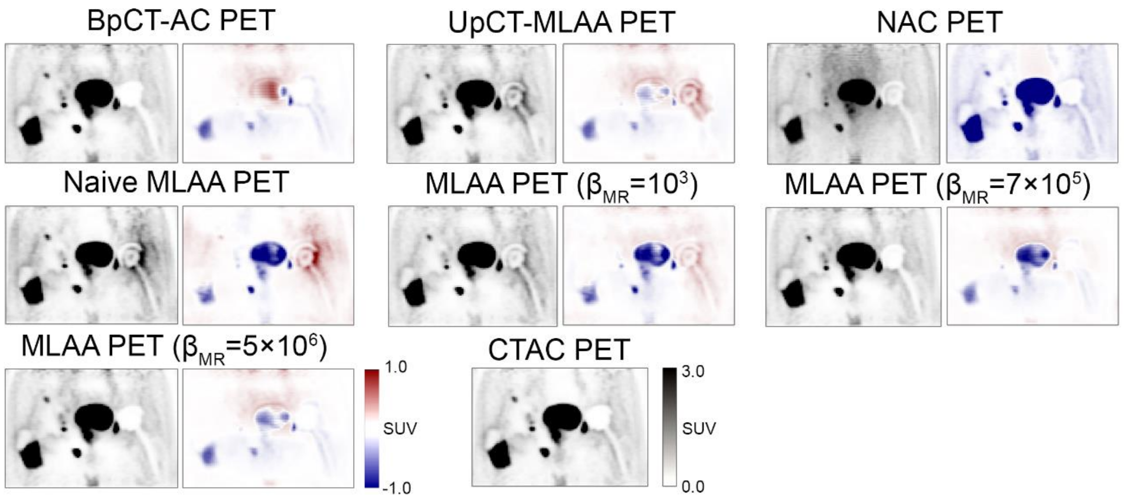

Supp. Fig. 8. PET images and difference images for a patient with a metal implant imaged with PSMA.

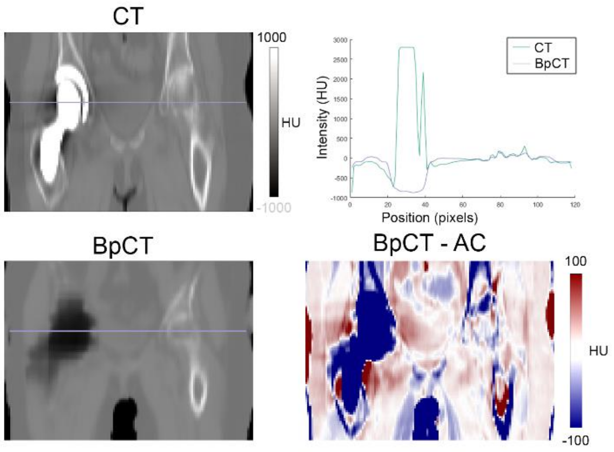

Supp. Fig. 9. CT, pseudo-CT, line profiles, and difference images for a patient with a metal implant imaged with FDG.

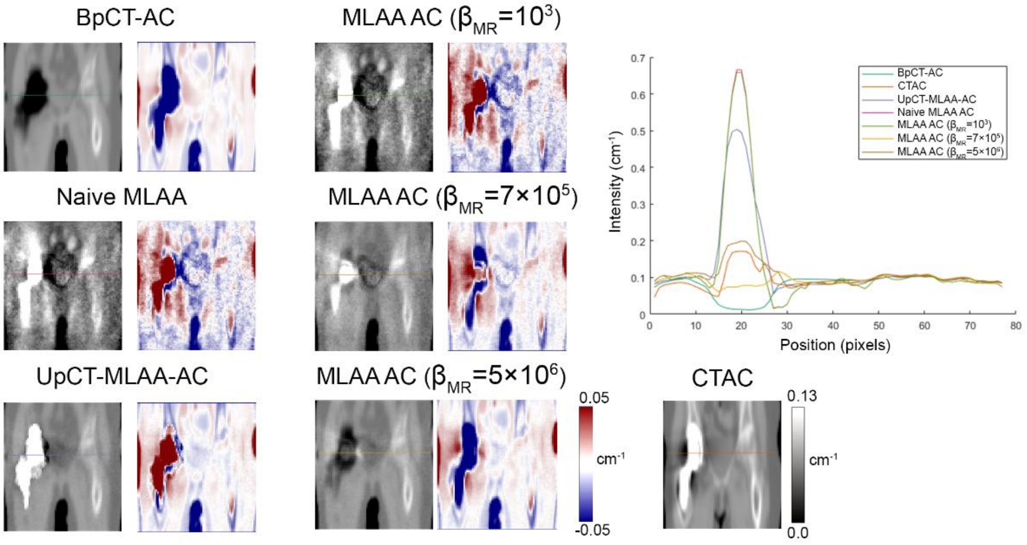

Supp. Fig. 10. AC maps, line profile, and difference images for a patient with a metal implant imaged with FDG.

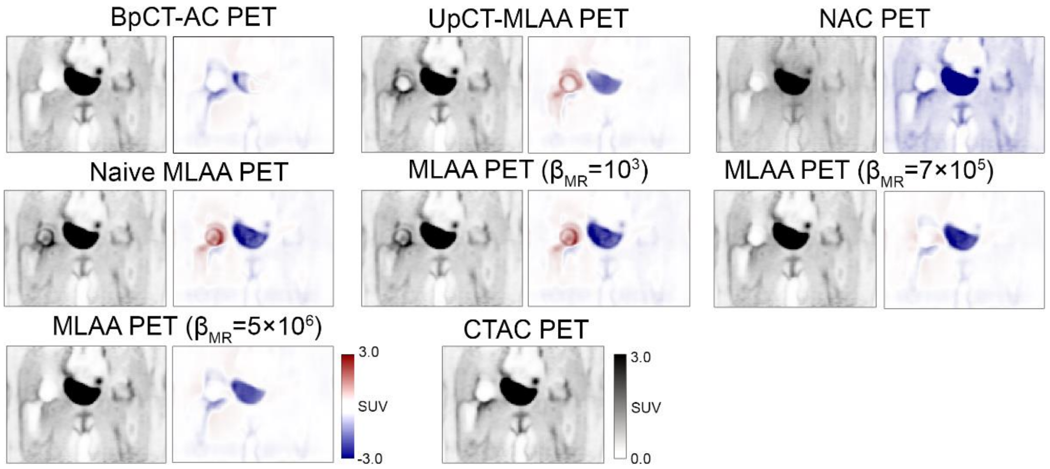

Supp. Fig. 11. PET images and difference images for a patient with a metal implant imaged with FDG.
